# CMIC: predicting DNA methylation inheritance of CpG islands with embedding vectors of variable-length *k*-mers

**DOI:** 10.1186/s12859-022-04916-3

**Published:** 2022-09-12

**Authors:** Osamu Maruyama, Yinuo Li, Hiroki Narita, Hidehiro Toh, Wan Kin Au Yeung, Hiroyuki Sasaki

**Affiliations:** 1 Faculty of Design, Kyushu University, Fukuoka, Japan; 2 Graduate School of Design, Kyushu University, Fukuoka, Japan; 3 School of Design, Kyushu University, Fukuoka, Japan; 4 Division of Epigenomics and Development, Medical Institute of Bioregulation, Kyushu University, Fukuoka, Japan

**Keywords:** Recurrent neural network, Gated recurrent unit, Classification, Oocyte, Blastocyst, Embryo, Epigenetic modification, Reprogramming, Development

## Abstract

**Background:**

Epigenetic modifications established in mammalian gametes are largely reprogrammed during early development, however, are partly inherited by the embryo to support its development. In this study, we examine CpG island (CGI) sequences to predict whether a mouse blastocyst CGI inherits oocyte-derived DNA methylation from the maternal genome. Recurrent neural networks (RNNs), including that based on gated recurrent units (GRUs), have recently been employed for variable-length inputs in classification and regression analyses. One advantage of this strategy is the ability of RNNs to automatically learn latent features embedded in inputs by learning their model parameters. However, the available CGI dataset applied for the prediction of oocyte-derived DNA methylation inheritance are not large enough to train the neural networks.

**Results:**

We propose a GRU-based model called CMIC (CGI Methylation Inheritance Classifier) to augment CGI sequence by converting it into variable-length *k*-mers, where the length *k* is randomly selected from the range $k_{\min }$ to $k_{\max }$, *N* times, which were then used as neural network input. *N* was set to 1000 in the default setting. In addition, we proposed a new embedding vector generator for *k*-mers called splitDNA2vec. The randomness of this procedure was higher than the previous work, dna2vec.

**Conclusions:**

We found that CMIC can predict the inheritance of oocyte-derived DNA methylation at CGIs in the maternal genome of blastocysts with a high F-measure (0.93). We also show that the F-measure can be improved by increasing the parameter *N*, that is, the number of sequences of variable-length *k*-mers derived from a single CGI sequence. This implies the effectiveness of augmenting input data by converting a DNA sequence to *N* sequences of variable-length *k*-mers. This approach can be applied to different DNA sequence classification and regression analyses, particularly those involving a small amount of data.

**Supplementary Information:**

The online version contains supplementary material available at 10.1186/s12859-022-04916-3.

## Background

DNA methylation is an epigenetic modification that occurs primarily at CpG sites and regulates gene expression. Hence, DNA methylation significantly impacts various mammalian biological processes, including embryonic development, genomic imprinting, X-chromosome inactivation, repression of transposable elements, aging, and carcinogenesis. Global epigenetic reprogramming is an erasure process associated with remodeling of epigenetic modifications that occurs in primordial germ cells and early embryos, ensuring that a new developmental cycle begins in each generation [1]. A notable exception to global reprogramming in early embryos is genomic imprinting, in which differential DNA methylation established in parental gametes is transmitted to the zygote and regulates parental-origin-specific gene expression [2]. However, recent studies have shown that ectopic methylation induced by environmental factors may also escape reprogramming and cause disease susceptibility in subsequent generations [3].

CpG islands (CGIs) are CpG-dense regions in the genome that often overlap with gene promoters [4]. Approximately 16,000–22,000 CGIs have been identified within the mouse genome, most of which remain unmethylated throughout development. Alternatively, certain CGIs become methylated in specific tissues or are predisposed to methylation under specific conditions, such as cell culture and carcinogenesis. CGIs can also be methylated upon genomic imprinting and X-chromosome inactivation [4]. However, in all cases, regardless of methylation status, most CpG sites within a CGI behave similarly, and their methylation strongly downregulates associated genes [4].

We have previously studied how DNA methylation introduced at CGIs, including imprinting control regions, is transmitted from oocytes to embryos [5–7]. In mouse oocytes, approximately 1100 CGIs are methylated via transcription-coupled de novo methylation [8]. CGI methylation is then erased after fertilization by reprogramming; however, a subset (up to 15%) of CGIs, including the maternally methylated imprinting control regions, can remain methylated in blastocysts [8]. This CGI methylation inheritance is partly dependent on the sequence-specific methylated-DNA-binding protein, Zfp57, which recruits other proteins essential for methylation maintenance [9]. Therefore, we focused on determining whether CGI sequences contain sufficient information to predict methylation inheritance through fertilization and early development. Previous studies have reported methods to predict CGI methylation status, or their propensity for methylation, of various cell types and tissues based on the sequence and other features [10–16]. Notably, Zheng *et al*. reported a support vector machine-based models that achieved high specificity and accuracy involving histone modification data (methylation and acetylation) [15]. However, it is difficult to obtain high-quality histone modification information from oocytes and blastocysts, due to the scarcity of samples and ethical issues (especially when involving humans).

Recurrent neural network (RNN)-based approaches have advantages over the above-mentioned methods for sequence-based methylation prediction. First, neural networks can automatically learn latent feature representations of input data without prior biological knowledge. In contrast, previous studies used decision trees, support vector machines, or logistic regression, which required previously-designed feature vectors as input. Second, RNNs take data of variable length as input, including sequences from CGIs of different sizes. RNN-based classifiers have been widely used in bioinformatics, for example in KEGRU, for the prediction of transcription factor binding sites [17], DNA sequence functions [18], and chromatin accessibility [19]. KEGRU divides DNA sequences into *k*-mer sequences with specified length and stride and, considering each *k*-mer as a word, converts them into pre-trained embedding vectors using the word2vec algorithm [20]. It then constructs a bidirectional gated recurrent unit (BiGRU) neural network for classification. Although KEGRU is applicable to our experimental aim, one challenge remains. From a pool of CGIs that are methylated in oocytes, we selected those that could be evaluated in methylated or unmethylated form in the maternal genome of blastocysts using single nucleotide polymorphism (SNP) information. However, this process resulted in only 272 CGIs, which is not sufficient to train RNNs.

Hence, we designed CMIC (CGI Methylation Inheritance Classifier) as a new method of converting a CGI sequence into *k*-mer sequences. A CGI sequence is partitioned into variable-length *k*-mers such that neighboring *k*-mers do not overlap, which is repeated *N* times. The variable-length *k*-mer sequences share the methylation status of a CGI as a class label. Thus, we augmented the amount of input data given to the GRU network. Furthermore, the variable-length *k*-mer sequences from the entire set of informative CGIs were also used to create pre-trained embedding vectors using the word2vec algorithm, and this new embedding assignment method is called splitDNA2vec. The sequence of the embedding vectors is passed to a BiGRU layer to predict the DNA methylation status of the input sequence, which we designated as CGI methylation classification method CMIC.

We show that CMIC achieves a high F-measure of 0.93. We also discuss how the value of *N* affects the prediction results of the CMIC. Taken together, this work demonstrates that converting a CGI sequence into multiple variable-length *k*-mer sequences is effective for predicting its DNA methylation status.

## Methods

**Fig. 1:**
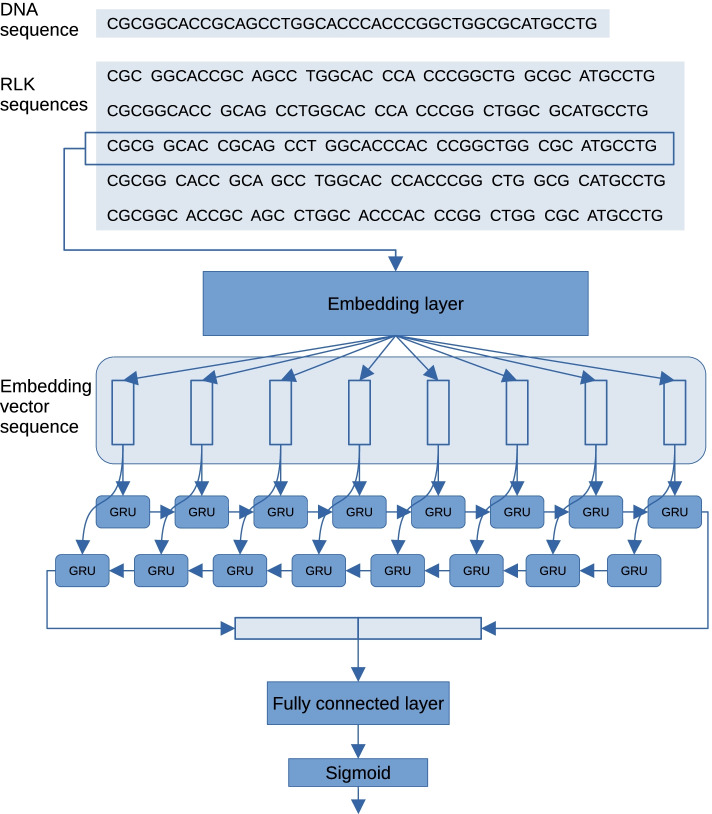
Framework of CMIC. Given a DNA sequence *s*, multiple variable-length *k*-mer sequences of *s* are generated first. The input layer of the neural network takes a variable-length *k*-mer sequence of as the input. The second layer is an embedding layer in which each *k*-mer of the inputted sequence is in order mapped to the corresponding embedding vector. The initial embedding vectors in the embedding layer are created using our new embedding vector generation method called splitDNA2vec. The third layer is a BiGRU layer comprising forward and backward directional GRU neural networks. The two outputs of the previous layer are concatenated into a single vector, and inputted into the next fully connected layer followed by a sigmoid activation function. The output is interpreted as an estimation of the probability that *S* is unmethylated

**Fig. 2:**
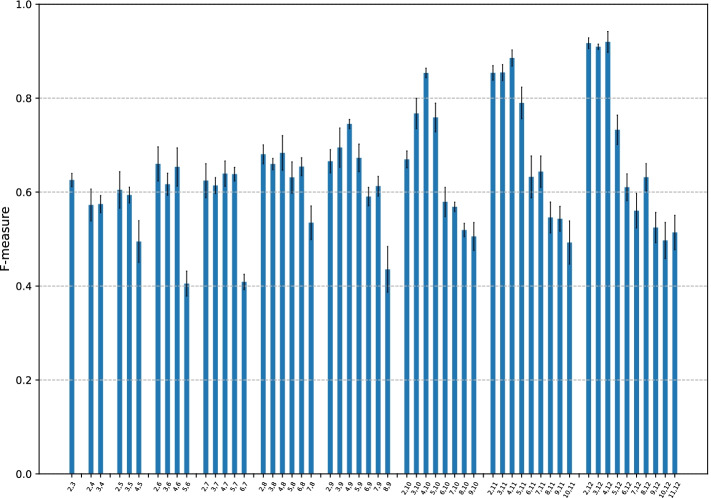
F-measure of CMIC with different pairs of $k_{\min }$ and $k_{\max }$. The search space of the pairs of $k_{\min }$ and $k_{\max }$ are set to be $k_{\min } = 2, \ldots , 11$ and $k_{\max } = 3, \ldots , 12$ with $k_{\max } - k_{\min } \ge 1$. The bars are grouped by $k_{\max }$

**Fig. 3:**
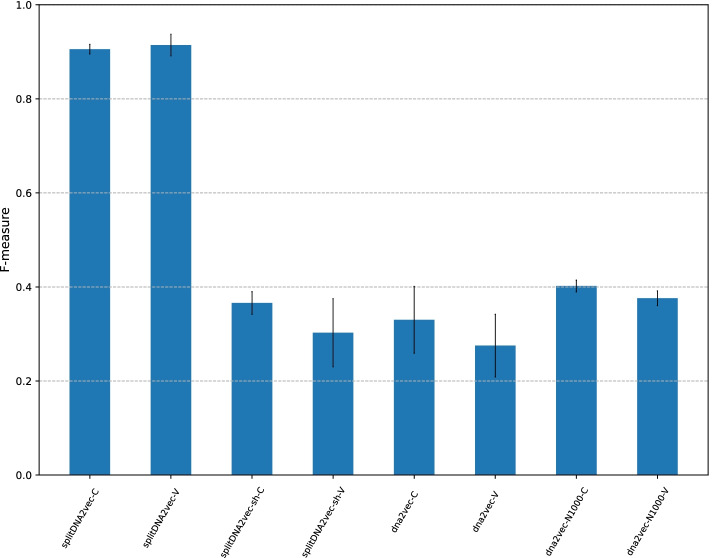
F-measure with different schemes for embedding vectors shown in Table 3. The x-axis represents the eight methods to be compared: splitDNA2vec-C, splitDNA2vec-V, splitDNA2vec-sh-C, splitDNA2vec-sh-V, dna2vec-C, dna2vec-V, dna2vec-N1000-C, and dna2vec-N1000-V. The y-axis shows the F-measure on test datasets

**Fig. 4:**
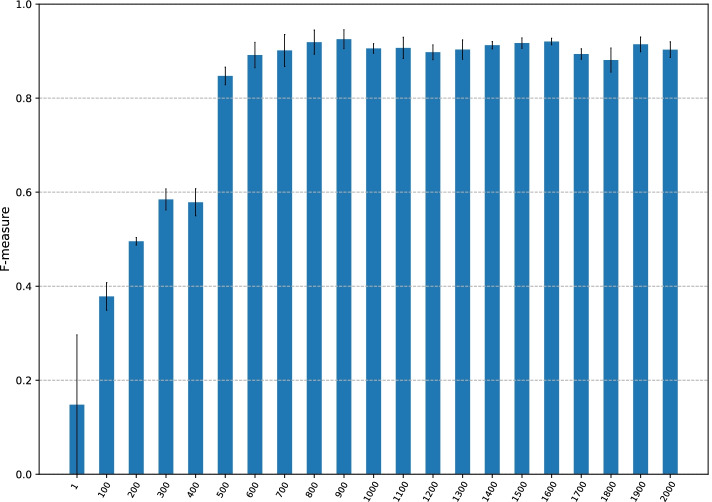
F-measure of CMIC with different numbers of variable-length *k*-mer sequences generated from an input CGI sequence, *N*. The x-axis represents values of *N*. The y-axis shows the F-measure with *N*

**Fig. 5:**
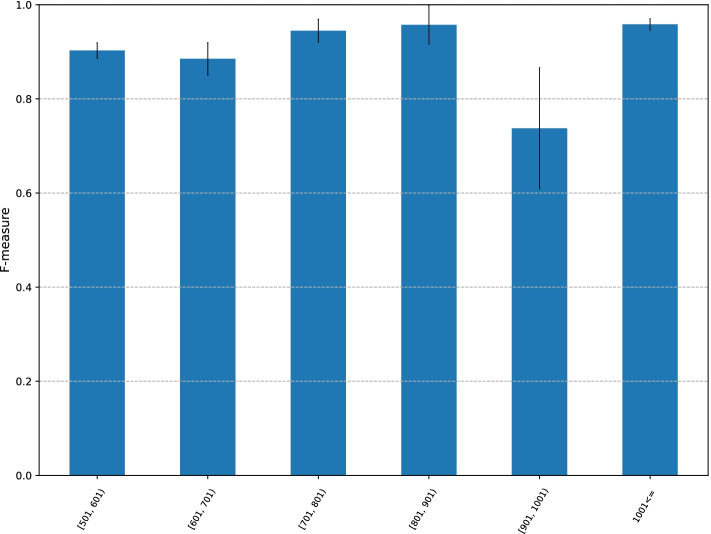
F-measure of CMIC trained with short CGIs for long CGIs. The x-axis indicates the range of CGI sequence lengths. The y-axis indicates the F-measure

**Fig. 6:**
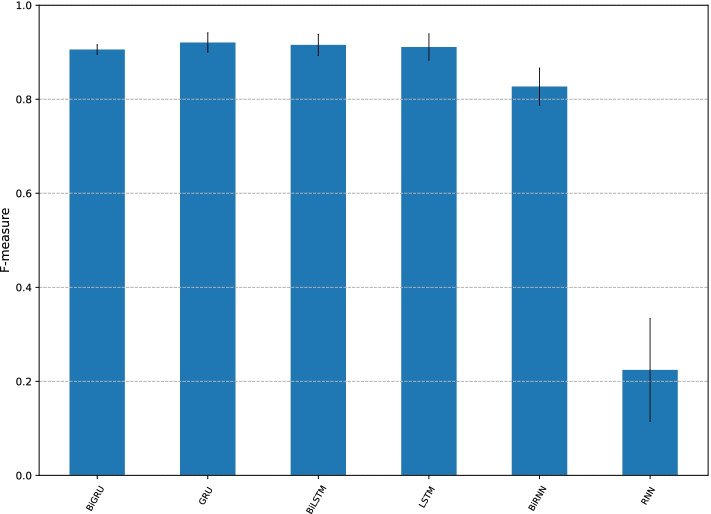
F-measure of CMIC with alternative recurrent units, RNN, BiRNN, GRU, BiGRU, LSTM, and BiLSTM

**Fig. 7:**
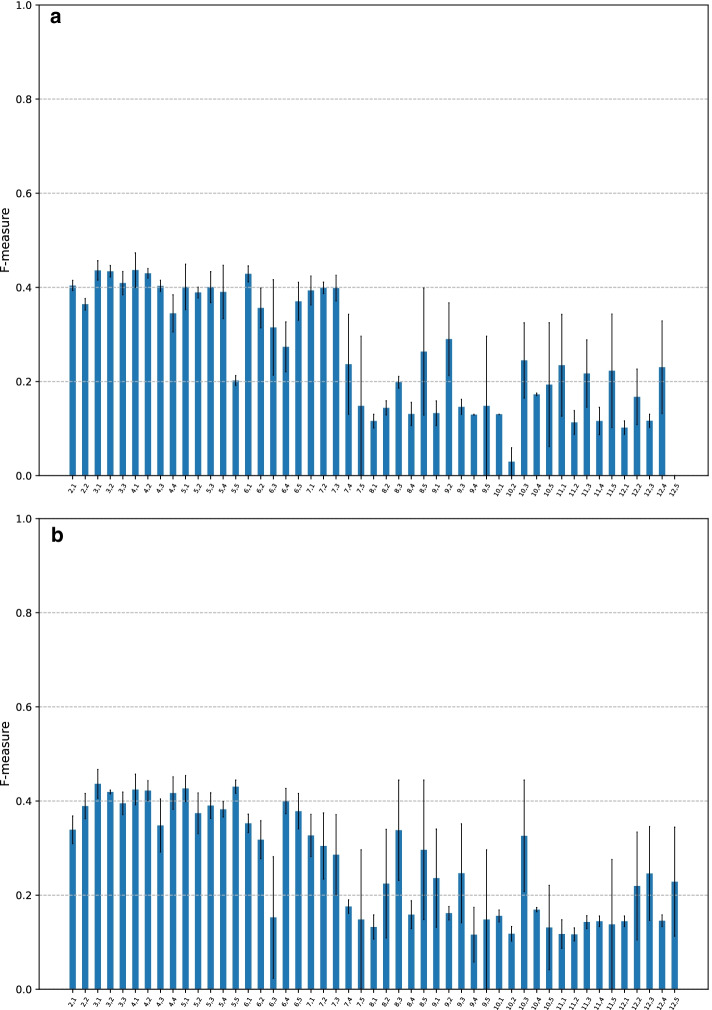
F-measure of KEGRU with various lengths of *k*-mers and strides. The x-axis represents a pair of *k* and stride. **a** $D=20$. **b** $D=50$

**Fig. 8:**
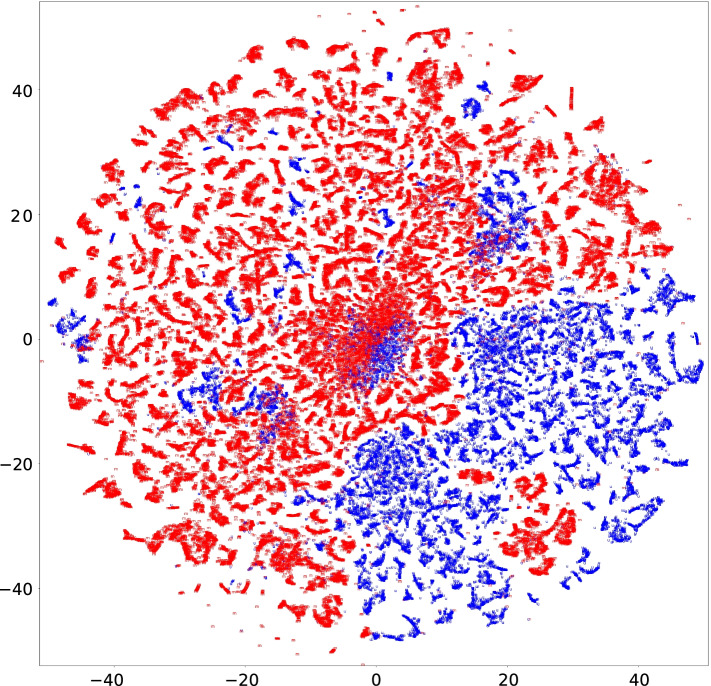
Output of t-SNE. Each point corresponds to a 12-mer. The point of a 12-mer extracted from unmethylated (methylated, resp.) CGI sequences is represented as u (m, resp.)

An outline of our proposed method CMIC is as follows (see Fig. 1). When CMIC takes a DNA sequence *s* as input, 2*N* variable-length *k*-mer sequences are generated by splitting *s* and its reverse complement into *k*-mers where such a length *k* is determined at random according to the discrete uniform distribution on an interval. Each variable-length *k*-mer sequence is taken as the input to the BiGRU neural network of CMIC. The *k*-mers of a given variable-length *k*-mer sequence are converted to the corresponding embedding vector in the embedding layer. Next, the resulting sequence of embedding vectors is given to the BiGRU layer. The output to the input variable-length *k*-mer sequence is interpreted as the probability of *s* being unmethylated.

### Generating sequences of random-length *k*-mers from DNA sequences

In the first step of CMIC, an input DNA sequence *s* is split into non-overlapping *k*-mers with lengths randomly determined according to a discrete uniform distribution on the interval from a minimum to a maximum length, $k_{\min }$ and $k_{\max }$, respectively. This step is repeated *N* times for each *s* and its reverse complement. As a result, 2*N* variable-length *k*-mer sequences are generated from the same *s* sequence. These sequences are referred to as cognate because the source DNA sequence is the same. These variable-length *k*-mer sequences are used as inputs to the BiGRU neural network of CMIC. This process is a data augmentation method as the number of input instances is increased (see, for example, [21]).

Furthermore, such multiple cognate variable-length *k*-mer sequences are alternative views of the original DNA sequence. Thus, multiple cognate variable-length *k*-mer sequences are expected to make the trained neural network more robust.

### Neural network architecture

We here describe the design of the BiGRU neural network of CMIC. As mentioned in the previous section, the input layer takes a variable-length *k*-mer sequence as input. The second layer of the network is an embedding layer in which each *k*-mer of the given sequence is converted to the corresponding real vectors of dimension *D*. We initialized the embedding vectors of the layer with the embedding vectors generated by our embedding vector generation method, splitDNA2vec, described in the subsequent subsection. The resulting embedding vector sequences are passed to the BiGRU layer, where they are processed in the forward and backward direction. The GRU at each time in each direction holds a hidden state represented by a *H*-dimensional vector. The last hidden states of the forward and backward directions are concatenated into a single vector. This vector is inputted into a fully connected layer followed by a sigmoid activation function. The output of the function is the total output of this network, and is interpreted as the probability of the input being unmethylated.

A training dataset is a pair of variable-length *k*-mer sequence $x_i$ and its methylation status $y_i \in \{0,1\}$ for $i = 1, \ldots , M$, where $y_i = 1$ (0, resp.) indicates unmethylated (methylated, resp.). We denote the parameters of the neural network of CMIC by ${\mathbf {w}}$. Let ${\mathbf {y}} = (y_1, \ldots , y_M)$ and $\hat{{\mathbf {y}}} = ({\hat{y}}_1, \ldots , {\hat{y}}_M)$ be the observed (true) and predicted methylation statuses of $x_1, \ldots , x_M$. In the process of training the model, the model parameter ${\mathbf {w}}$ is optimized with the training dataset by minimizing the loss function

$$\begin{aligned} L({\mathbf {w}}) = E(\hat{{\mathbf {y}}}, {\mathbf {y}} ) + \alpha \cdot \Vert {\mathbf {w}} \Vert _2 \end{aligned}$$

where $\alpha$ is the weight decay hyper-parameter, $\Vert w\Vert _2$ is the $L_2$ norm, and $E(\hat{{\mathbf {y}}}, {\mathbf {y}} )$ is the cross-entropy function, defined as

$$\begin{aligned} E(\hat{{\mathbf {y}}}, {\mathbf {y}} ) = -\sum _{i=1}^M (y_i\log \hat{y_i}+(1-y_i)\log (1-\hat{y_i})). \end{aligned}$$

In addition to the embedding vector dimension *D*, GRU hidden vector dimension *H*, and weight decay rate $\alpha$, there are several hyper-parameters of the BiGRU aspect of CMIC used in the learning process namely, epoch, *e*, learning rate, *r*, batch size, *b*, and dropout rate, *d*. The dropout technique is applied in the BiGRU layer [22]. Their default values are given in Table 1. We train the network by Adaptive Moment Estimation (Adam) [23], a stochastic gradient descent optimization algorithm.

**Table 1:** Default values of hyper-parameters of CMIC.

Symbol	Default value	Description
*N*	1000	Number of variable-length *k*-mer sequences generated from a CGI sequence
*D*	20	Embedding vector dimension
*H*	256	Hidden variable dimension
*e*	2	Epoch
*r*	0.0001	Learning rate
*b*	32	Batch size
*d*	0.5	Dropout rate
$\alpha$	0.01	Weight decay rate

These values are used without explicit mention of the parameters

**Table 2:** Threshold setting for cell-types.

Sample	*T*	$\beta _{M}$	$\beta _{U}$
FGO	100	0.8	0.1
maternal genome of blastocysts	20	0.4	0.1

*T* is the lower bound for the whole read count of a CGI which is used in analysis. $\beta _{M}$ is the lower bound for the methylation ratio of a CGI which belongs to class M (methylated), $\beta _{U}$ is the upper bound for the methylation ratio of a CGI which belongs to class U (unmethylated)

**Table 3:** Methods of generating variable-length *k*-mers.

Method	*k*-mer overlap	Vector assignment
splitDNA2vec	no	word2vec
splitDNA2vec-sh	no	random shuffling of the word2vec map from *k*-mers to embedding vectors
dna2vec	yes	word2vec

Each column shows the name of a method, overlapping between adjacent variable-length *k*-mers, and the method used for assigning vector elements

**Table 4:** Distribution of long CGI lengths. Column label “*L*-” indicates the sequence length range $$[L, L+100)$$ for $$L=501, \ldots , 901$$, and 1001- is sequences of length 1001 and more

Class	501-	601-	701-	801-	901-	1001-
M2M	9	8	3	2	3	7
M2U	10	7	3	2	1	7

**Table 5:** Hyper-parameter setting of KEGRU.

hyper-parameter	[17]	this study
*k*	4, 5, 6	$2, \ldots , 12$
stride	$2, \ldots , 5$	$1, \ldots , 5$
*D*	50, 100, 150, 200	20, 50

*k* represents the length of *k*-mer. In [17], they compared the cases where $D = 50, 100, 150$, and 200, and reported almost the same scores with $D = 50, 100$, and 150. Thus, we adopted $D=50$ as well as our default value $D=20$

**Table 6:** Prediction result on the DNA methylation of CGIs in human lymphocytes

Method	balanced accuracy	F-measure	MCC
CMIC	0.949	0.895	0.866
Bock *et al.*	0.830	0.784	0.752
Ratio (%)	14	14	15

### splitDNA2vec: generator of embedding vectors of random-length *k*-mers

Here, we formulate our new embedding vector creation method, called *splitDNA2vec*. The embedding vectors in the embedding layer of CMIC are initialized with the output of this method. First, variable-length *k*-mer sequences are generated using the same procedure as in the first step described for CMIC with $N=1000$ from all available CGIs, which are equivalent to training and test data. The generated variable-length *k*-mer sequences are inputted into the word2vec algorithm with the continuous bag-of-words (CBOW) model. The parameter of the word2vec algorithm is configured as follows. The context window size is set to 10. The minimal count of a word, *i.e.*, the *k*-mer, is $c_{\min } = 1$. Namely, all occurrences of *k*-mers are counted. The dimension of embedding vectors is set to $D=20$.

This process of generating 2*N* variable-length *k*-mer sequences from a DNA sequence is inspired by dna2vec [24] in which a given DNA sequence is repeatedly segmented into overlapping *k*-mers, extracted from a sliding window of striding one, whose lengths are randomly chosen according to a discrete uniform distribution. The major difference between the dna2vec and our method, splitDNA2vec, is that our variable-length *k*-mer sequences have higher randomness than those of dna2vec because the length and the start position of an extracted *k*-mer are both random in our method, while only the length of *k*-mers are randomized in dna2vec. Furthermore, splitDNA2vec generates *N* sequences of variable-length *k*-mers from the same DNA sequence, though dna2vec generates only one sequence of variable-length *k*-mers from a DNA sequence.

In place of splitDNA2vec, we also evaluated the case where the embedding layer is initialized with the dna2vec algorithm output. Furthermore, we considered a shuffled version of splitDNA2vec, denoted by splitDNA2vec-sh, in which the mapping of *k*-mers to embedding vectors made by splitDNA2vec is randomly shuffled. Finally, we examined how these embedding vector initialization methods affect performance of CMIC.

### Gated recurrent unit

In this section, we explain the BiGRU neural network used in CMIC. The GRU was formulated by Cho *et al.* [25], and is similar to a long short-term memory (LSTM) [26], but simpler as it does not use an output gate. We discuss performance of the CMIC variants with the standard RNN and LSTM architectures in the result section.

We here explain the architecture of GRU. The reset gate takes as input the input at time step *t*, ${\mathbf {x}}^t$, and the output (hidden) vector at time step $t-1$, ${\mathbf {h}}^{t-1}$. This gate outputs a reset gate vector, ${\mathbf {r}}_t$, given as

$$\begin{aligned} {\mathbf {r}}^t = \sigma ({\mathbf {W}}_r {\mathbf {x}}^t + {\mathbf {U}}_{r} {\mathbf {h}}^{t-1} + {\mathbf {b}}_r) \end{aligned}$$

where ${\mathbf {W}}_r$ and ${\mathbf {U}}_{r}$ are weight matrices for ${\mathbf {x}}^t$ and ${\mathbf {h}}^{t-1}$, respectively, ${\mathbf {b}}_r$ is a bias, and $\sigma$ is a sigmoid function. Similarly, the update gate outputs an update gate vector, ${\mathbf {z}}_t$, defined as

$$\begin{aligned} {\mathbf {z}}^t = \sigma ({\mathbf {W}}_z {\mathbf {x}}^t + {\mathbf {U}}_{z} {\mathbf {h}}^{t-1} +{\mathbf {b}}_z). \end{aligned}$$

${\mathbf {W}}_z$ and ${\mathbf {U}}_{z}$ are weight matrices for ${\mathbf {x}}^t$ and ${\mathbf {h}}^{t-1}$, respectively, and ${\mathbf {b}}_z$ is a bias.

Using ${\mathbf {r}}^t$ as a regulator, GRU generates $\tilde{{\mathbf {h}}}^t$ the candidate activation vector at time step *t*, calculated as

$$\begin{aligned} \tilde{{\mathbf {h}}}^t = tanh ({\mathbf {W}} {\mathbf {x}}^t + {\mathbf {U}} ( {\mathbf {r}}^t \odot {\mathbf {h}}^{t-1} ) +{\mathbf {b}}_h ) \end{aligned}$$

where ${\mathbf {W}}$ and ${\mathbf {U}}$ are weight matrices, ${\mathbf {b}}_h$ is a bias, and $\odot$ is the Hadamard product. Note that $tanh$ is the hyperbolic tangent function, whose range is $[-1,1]$. Further, the reset gate vector ${\mathbf {r}}^t$ is used as a coefficient representing how much the output vector, ${\mathbf {h}}^{t-1}$, should be forgotten to make the candidate activation vector, $\tilde{{\mathbf {h}}}^t$.

The output vector at time step *t*, ${\mathbf {h}}^t$, is the affine combination of ${\mathbf {h}}^{t-1}$ and $\tilde{{\mathbf {h}}}^{t}$ with a ratio of ${\mathbf {z}}^t : ({\mathbf {1}} - {\mathbf {z}}^t )$; that is,

$$\begin{aligned} {\mathbf {h}}^t = {\mathbf {z}}^t \odot {\mathbf {h}}^{t-1} + ({\mathbf {1}} - {\mathbf {z}}^t) \odot \tilde{{\mathbf {h}}}^{t}, \end{aligned}$$

where ${\mathbf {1}}$ is the vector filled with ones of the same dimension as ${\mathbf {z}}^t$.

To enhance the predictability of CMIC, we use the BiGRU model, which is the GRU version of the BiRNN model [27]. Namely, it has forward and backward GRU networks, where an input is processed in the forward and backward directions, respectively, which we call BiGRU layer. The output of a BiGRU layer is formulated by merging the last hidden state vectors of two directions with an appropriate mode, like concatenation, summation, average, and multiplication. Concatenation is adopted in this work.

### Materials

We use the data obtained by whole-genome bisulfite sequencing (WGBS) of mouse fully grown oocytes (FGOs) [6, 7, 28, 29] and blastocyst (GSE174311). The FGOs are obtained from the mouse strain C57BL/6J (Kyudo Co, Japan), and the blastocysts are derived from in vitro fertilization of C57BL/6J oocytes with JF1/Ms mouse (Genetic Resource Center, National Institute of Genetics, Japan) sperm. At the time of the experiments the mice were at least 10 weeks old. The locations of CGIs are based on the mm10 assembly of the mouse genome and obtained from the UCSC genome annotation database [30]. The WGBS reads of the FGO and blastocyst were mapped to the mouse genome using Bismark [31].

For allelic-specific methylation analysis of blastocysts, reads were mapped to an N-masked genome sequence generated based on the published SNP data of JF1 [32]. Allelic reads were selected using SNPsplit [33].

### Methylation status of CGIs

For each CGI, we count the number of WGBS reads covering a CpG site within the CGI and that of reads represented as methylated. We define the DNA methylation ratio of a CGI as the count for methylation to the whole count. If the whole read count of a CGI is less than the threshold *T*, it is not used in any further analysis. Then, a CGI is labeled M (methylated) if its methylation ratio is greater than or equal to a threshold $\beta _{M}$, while it is labeled U (unmethylated) if its methylation ratio is lower than a threshold, $\beta _{U}$. Table 2 shows the setting of these parameters for FGO and maternal genome of blastocyst. The distribution of methylation ratios in the maternal genome of mouse blastocysts of the CGIs that are methylated in FGOs is given in Additional file 1: Fig. S1.

Our target data is the methylation status, either M or U, of CGIs that belongs to class M in FGOs, in the maternal genome of blastocyst. These classes are respectively denoted as M2M (DNA methylation inheritance) and M2U (DNA methylation loss). The number of M2M CGIs is 182, and that of M2U CGIs is 90. Among them, there are 150 M2M CGIs and 60 M2U CGIs with lengths of at most 500 bp. These CGIs are suitable for learning models because longer sequences require much more time to train models. We use them in 3-fold stratified cross-validation.

### Performance metrics

The output of CMIC to an input variable-length *k*-mer sequence is the probability of the input being unmethylated. We determine the predicted class label as “unmethylated” if the probability is greater than 0.5 or “methylated” otherwise.

As a performance metrics, we use balanced accuracy, F-measure, MCC (Matthews correlation coefficient), and AUC. We calculate these performance metrics in 3-fold stratified cross-validation and show the average with the standard error.

## Results

### Finding the best pair of the lower and upper bounds of the variable length of extracted *k*-mers

Recall that $k_{\min }$ and $k_{\max }$ are the lower and upper bounds of the variable-length *k*-mers which a given DNA sequence are split into. We here find the best pair of $k_{\min }$ and $k_{\max }$ with the other default parameters. We set the search space of the pairs of $k_{\min }$ and $k_{\max }$ such that $k_{\min } = 2, \ldots , 11$ and $k_{\max } = 3, \ldots , 12$ such that $k_{\max } - k_{\min } \ge 1$.

The F-measure based on a 3-fold cross-validation is summarized in the bar graph in Fig. 2 where bars are grouped by $k_{\max }$. Results show that as $k_{\max }$ increases, so too do the F-measure values with a small $k_{\min }$ value of approximately 2, 3, and 4. Given that splitDNA2vec assigns the embedding vectors of such short and longer *k*-mers simultaneously, these embedding vectors likely acted synergistically to characterize CGI sequences. Among them, $(k_{\min }, k_{\max }) = (4,12)$ achieved the highest F-measure, 0.93. This pair of values was used as the default in subsequent analyses. The balanced accuracy, MCC, and AUC graphs appear similar to that in Fig. 2 (Additional File 1: Figs. S2, S3, and S4).

Lastly, in the case of $k_{\max }$ values greater than 12, considering that CMIC marked the highest F-measure with $k_{\max } = 12$, we ran splitDNA2vec with $k_{\max } = 13$; however, this process was incredibly time consuming, particularly in executing word2vec; Thus we have not examined this case in depth.

### Evaluation of splitDNA2vec

We next analyzed the extent to which the embedding vectors, used in the embedding layer, affect prediction of the CGI methylation status. For this purpose, we considered three different methods for generating embedding vectors, as shown in Table 3. The first method was our default scheme, splitDNA2vec, the second method was splitDNA2vec-sh, which randomly shuffles the mapping of *k*-mers to embedding vectors made by splitDNA2vec, and the final method was dna2vec, which has been described above. Recall that dna2vec generates a sequence of variable-length *k*-mers from a DNA sequence. Here we extended dna2vec which generates 1000 sequences of variable-length *k*-mers from the same sequence. This extension is denoted by dna2vec-N1000.

This embedding vector assignment to *k*-mers is an unsupervised learning process. Thus, the DNA sequences of our dataset, including training, test, and long CGI datasets, were applied without their class labels. Note that each method generates $N=1,000$ variable-length *k*-mer sequences from an input CGI sequence, save for the original dna2vec.

We further formulated more specific embedding vector methods derived from each of the four methods described above, from the view point of whether the embedding vectors in the embedding layers are updated in the training process of the neural network part of CMIC, as in [34]. More specifically, these vectors were treated as variable weights of the network (V) or constant vectors (C). For splitDNA2vec, we denoted the two versions as splitDNA2vec-V and splitDNA2vec-C, and the remaining versions in the same way. Thus, we compared the resulting eight methods with the remaining parameters as default.

The F-measure on the test datasets of the eight different options explained above are shown in Fig. 3. This result has some interesting implications.

First, the best method was found to be splitDNA2vec-V with an F-measure of 0.914, followed by splitDNA2vec-C with an F-measure of 0.906. This implies that even if the embedding layer is freezed, CMIC can achieve same-level performance due to the effectiveness of the embedding vectors constructed by the splitDNA2vec method. Moreover, the splitDNA2vec-C method offers the advantage of not requiring further updates of the initial embedding vectors given by the splitDNA2vec method in the process of end-to-end training of the CMIC neural network. Thus, we use splitDNA2vec-C as the default setting in further analyses.

Second, we can see that dna2vec-N1000-C and dna2vec-N1000-V are superior to dna2vec-C and dna2vec-V, highlighting the benefit of generating multiple sequences of variable-length *k*-mers from the same CGI sequences. The highest F-measure obtained was 0.40, given by dna2vec-N1000-C, which is significantly lower than the 0.91 of splitDNA2vec. Given that the primary difference between splitDNA2vec and dna2vec-N1000 is the presence/absence of overlap between neighboring extracted *k*-mers, this result implies that overlapping neighboring *k*-mers have less information than non-overlapping ones.

Third, in terms of the F-measure, dna2vec-C and dna2vec-V yielded worse results than the randomly-shuffled version of splitDNA2vec, splitDNA2vec-sh-C and splitDNA2vec-sh-V, respectively. The option “window” of word2vec, specifying the length of consecutive *k*-mers used as an instance to train the word2vec model, was set to 10 in all eight methods for embedding vectors. A possible drawback of dna2vec is that the genomic region covered by a sliding window of size 10 is shorter than splitDNA2vec due to overlapping between neighboring *k*-mers.

The graphs of balanced accuracy, MCC, and AUC are similar to Fig. 3 (given in Additional file 1: Figs. S5, S6, and S7).

### The number of variable-length *k*-mer sequences strongly affects DNA methylation inheritance prediction

The total number of class M2U CGIs is 60, which may not be sufficient to adequately train CMIC. We then increased *N*, the number of sequences of variable-length *k*-mers generated from an input CGI sequence, which are directly taken as input for the network. The result is shown in Fig. 4.

As *N* increases to 900, the F-measure gradually improved and attained an F-measure of 0.93 at $N=900$. Subsequently, the curve plateaued. This result clearly indicates that multiple sequences of variable-length *k*-mers should be used to train models.

The graphs of balanced accuracy, MCC, and AUC are similar to Fig. 4 (given in Additional file 1: Figs. S8, S9, and S10).

### Predictability of the trained model for long CGIs

In this work, we limited the length of CGIs in training datasets to 500 bp as longer sequences cause the unfolded form of the CMIC neural network to be deeper and make the learning process more difficult. If a key feature for determining the methylation inheritance of a CGI was encoded in a relatively short region, the network trained with the short CGIs could learn the feature and accurately predict the methylation inheritance of CGIs longer than 500 bp with the same level predictability. We then examined the F-measure of the trained CMIC with the short sequences for the longer ones.

The length distribution of long M2M and M2U CGIs is given in Table 4. The F-measure of prediction on these sequences is given in Fig. 5.

The average F-measure for the CGI length intervals [501, 600], [601, 700], [701, 800], [701, 800], [801, 900], 1000 and more are 0.90, 0.89, 0.94, 0.96, 0.74, and 0.96, respectively. Recall that, to this point, the best F-measure with the default parameter values was 0.93. Unexpectedly, three intervals attained F-measure values higher than 0.93. This result implies that some genomic features for methylation inheritance are embedded within regions of 500 bp or shorter, and are shared by many long CGIs.

The graphs of balanced accuracy, MCC, and AUC are similar to Fig. 5 (given in Additional file 1: Figs. S11, S12, and S13).

### Performance with different recurrent neural network architectures

Though the recurrent unit architecture adopted in CMIC is BiGRU in the default setting, there are other well-known architectures, including the standard RNN unit [35] and LSTM unit [26]. We then consider the variants of CMIC, in which the BiGRU network is replaced with the BiRNN network and the bidirectional LSTM (BiLSTM) network. Furthermore, we considered their unidirectional version, denoted by GRU, RNN, and LSTM.

Their F-measures are shown in Fig. 6. First, BiGRU, GRU, BiLSTM, and LSTM have comparable F-measures, which are higher than those of BiRNN and RNN. Hence, the bidirectionality in the GRU and LSTM architectures is not necessary. This implies that the genomic features for DNA methylation status of CGIs can be learned in one direction. Meanwhile, RNN showed poor performance, however, the bidirectional version, BiRNN, compensated for the drawback of RNN to some extent.

The graphs of balanced accuracy, MCC, and AUC are similar to Fig. 6 (given in Additional file 1: Figs. S14, S15, and S16).

### Performance of KEGRU

Among many neural network-based classifiers for variable-length DNA sequences, the model most similar to CMIC is KEGRU [17] in which with a fixed *k* and a fixed stride, a single sequence of *k*-mers is generated from a DNA sequence. Indeed, their scheme is essentially the same as dna2vec. This differs from CMIC, in which the generation of multiple sequences of variable-length *k*-mers is performed by splitDNA2vec.

We then carried out comprehensive computational experiments of KEGRU with the hyper-parameter values shown in Table 5. We extended the range of *k*-mer lengths from the original lengths, 4, 5, 6, in [17] to $2, \ldots , 12$. We also widened the strides range from $2, \ldots , 5$ to $1, \ldots , 5$. Lastly, the dimensions of embedding vectors, denoted by *D*, were changed from 50, 100, 150, 200 to 20 and 50. In [17], the cases where $D = 50, 100, 150$, and 200 were compared, revealed relatively similar scores with those for $D = 50, 100$, and 150. Thus, we adopted $D=50$ in addition to our default value $D=20$.

Fig. 7a, b presents the F-measure graphs for KEGRU with $D=20$ and 50, respectively. Broadly speaking, the results are similar with the bars on the left axis showing F-measures of approximately 0.4, and those on the right, indicating approximately 0.2, some of which had large standard errors. A comparison of these results with those shown in Fig. 2 indicates the effectiveness of the multiple sequence generation of *k*-mers of splitDNA2vec.

The graphs of balanced accuracy, MCC, and AUC are similar to Fig. 7 (given in Additional file 1: Figs. S17, S18, S19, S20, S21, and S22).

### Characterizing embedding vectors of 12-mers

When we generate a sequence of variable-length *k*-mers from a CGI sequence using splitDNA2vec, the maximum length is set to be $k_{\max } = 12$. However, for example, the work on chromatin accessibility [19] used 6-mers, and KEGRU adopted $k= 4, 5$, and 6 as mentioned above for [17]. The reason these relatively short *k*-mers were selected was to avoid overfitting due to long *k*-mers. However, a long *k*-mer extracted from a training CGI sequence may never occur in any of the test CGI sequences, including the reverse complements. Hence, such a long *k*-mer will be an obstacle to training the models.

However, extracted *k*-mers are transformed into pre-trained *D*-dimensional embedding vectors in CMIC. Even if the two long *k*-mers differ, yet their substrings are similar or the same, the long *k*-mers may be mapped into embedding vectors close to each other. If this hypothesis is realized within methylated CGIs and/or unmethylated CGIs, such long *k*-mers can contribute to high predictability for CGI methylation inheritance.

To verify this hypothesis, we applied t-Distributed Stochastic Neighbor Embedding (t-SNE), which is an algorithm to map high-dimensional data into a low-dimensional space based on the distance between data points [36], to the 12-mers generated with the default parameter values of CMIC. Given that too many 12-mers were derived from CGI sequences of length $\le 500$, we removed the 12-mers occurring in both of methylated and unmethylated CGI sequences as they can not discriminate CGI methylation inheritance. A total of 107,129 12-mers remained. Their plot, generated by t-SNE with the default perplexity 30, is shown in Fig. 8. Most unmethylated and methylated 12-mers are respectively distributed into different regions according to their methylation inheritance. The plots with perplexity 10 and 50 show similar views (Additional file 1: Figs. S23 and S24). This implies that there are many 12-mers with embedding vectors characteristic of methylation inheritance.

### Predicting the methylation status of CGIs in human lymphocytes

In addition to our original DNA methylation inheritance prediction study aim, we also applied CMIC to the prediction of DNA methylation status of CGIs in human lymphocytes [37] used in Bock *et al.* [12], to evaluate the versatility of CMIC. The dataset contains 29 methylated and 103 unmethylated CGIs in chromosome 21. Given that this dataset is smaller than that comprising 60 M2U and 150 M2M CGIs, this study question is more challenging to CMIC.

As various attribute sets were tested, we selected one with the highest accuracy, 0.919, for this analysis. The attribute set is the combination of Class 1 (DNA sequence properties and patterns) and Class 2 (repeat frequency and distribution). They reported the total numbers of true positives (TPs), false negatives (FNs), true negatives (TNs), and false positives (FPs) over a 10-fold stratified cross-validation that was repeated 20 times (Table 2 in [12]). From this data we calculated balanced accuracy, F-measure, and MCC, shown in Table 6.

In the same way, we counted TPs, FNs, TNs, and FPs from the result on test datasets of 3-fold stratified cross-validation by CMIC, and gave the performance metrics in Table 6. As seen, CMIC marked 14% higher balanced accuracy and F-measure and 15 % higher MCC.

Note that Bock *et al*. designed a total of 1,184 sequence-based feature vectors and made predictions using support vector machines. Meanwhile, the key vectors of our method, which are embedding vectors of variable-length *k*-mers and hidden vectors of the BiGRU layer, are automatically learned by specifying hyper-parameters. This result indicates the superiority of CMIC.

## Conclusion

In this paper, we addressed whether it is possible to predict if CGIs maintain oocyte-derived methylation in the maternal genome of blastocysts, based on their sequences, and proposed a method, CMIC, for this prediction. A critical issue that arose was an insufficient number of available CGIs to train the neural networks. Thus, we designed a random data augmentation approach in which an input single DNA sequence was converted to multiple sequences of embedding vectors of extracted variable-length *k*-mers from the DNA sequence. Furthermore, such variable-length *k*-mers sequences derived from our CGI datasets were taken as input to generate embedding vectors of the *k*-mers. This new embedding vector generation method for DNA sequences was splitDNA2vec. These embedding vectors were used in the embedding layer of CMIC in the default setting. As a whole, CMIC takes an input CGI sequence, converts it to multiple variable-length *k*-mer sequences, and further transforms each *k*-mer to a specified embedding vector. A BiGRU network gives the probability of the input CGI inheriting the DNA methylation in the maternal genome of the blastocyst.

In this study, we found that splitDNA2vec works better than dna2vec for the methylation inheritance classification. Furthermore, we showed that the generation of numerous variable-length *k*-mer sequences from a DNA sequence is effective in augmenting input data, because the partition of a DNA sequence to variable-length *k*-mer sequences provides different representations of the original DNA sequences.

The design of the proposed method, CMIC, does not depend on DNA methylation. Thus, this method should be applicable to other DNA sequence classification problems including chromatin accessibility prediction [19].

## Supplementary Information

**Additional file 1. Fig. S1.** Distribution of methylation ratios in the maternal genome of mouse blastocyst of the CGIs that are methylated in FGOs. The lower bound of class M (methylated) for FGOs is set to *β*_M_ = 0.8 as shown in Table. 2.** Fig. S2.** Balanced accuracy of CMIC with different pairs of *k*_min_ and *k*_max_. The search space of the pairs of *k*_min_ and *k*_max_ are set to be *k*_min_ = 2, . . . , 11 and *k*_max_ = 3, . . . , 12 with *k*_max_ − *k*_min_ ≥ 1. The bars are grouped by *k*_max_. **Fig. S3.** MCC of CMIC with different pairs of *k*_min_ and *k*_max_. The search space of the pairs of *k*_min_ and *k*_max_ are set to be *k*_min_ = 2, ..., 11 and *k*_max_ = 3, ..., 12 with *k*_max_ − *k*_min_ ≥ 1. The bars are grouped by *k*_max_. **Fig. S4.** AUC of CMIC with different pairs of *k*_min_ and *k*_max_. The search space of the pairs of *k*_min_ and *k*_max_ are set to be *k*_min_ = 2, ..., 11 and *k*_max_ = 3, ..., 12 with *k*_max_ − *k*_min_ ≥ 1. The bars are grouped by *k*_max_. **Fig. S5.** Balanced accuracy with different schemes for embedding vectors shown in Table 3. The x-axis represents the eight methods to be compared: splitDNA2vec-C, splitDNA2vec-V, splitDNA2vec-sh-C, splitDNA2vec-sh-V, dna2vec-C, dna2vec-V, dna2vec-N1000-C, and dna2vec-N1000-V. The y-axis shows the F-measure on test datasets. **Fig. S6.** MCC with different schemes for embedding vectors shown in Table 3. The x-axis represents the eight methods to be compared: splitDNA2vec-C, splitDNA2vec-V, splitDNA2vec-sh-C, splitDNA2vec-sh-V, dna2vec-C, dna2vec-V, dna2vec-N1000-C, and dna2vec-N1000-V. The y-axis shows the F-measure on test datasets. **Fig. S7.** AUC with different schemes for embedding vectors shown in Table 3. The x-axis represents the eight methods to be compared: splitDNA2vec-C, splitDNA2vec-V, splitDNA2vec-sh-C, splitDNA2vec-sh-V, dna2vec-C, dna2vec-V, dna2vec-N1000-C, and dna2vec-N1000-V. The y-axis shows the F-measure on test datasets. **Fig. S8.** Balanced accuracy of CMIC with different numbers of variable-length *k*-mer sequences generated from an input CGI sequence, *N*. The x-axis represents values of *N*. The y-axis shows the F-measure with *N*. **Fig. S9.** MCC of CMIC with different numbers of variable-length *k*-mer sequences generated from an input CGI sequence, *N*. The x-axis represents values of *N*. The y-axis shows the F-measure with *N*. **Fig. S10.** AUC of CMIC with different numbers of variable-length *k*-mer sequences generated from an input CGI sequence, *N*. The x-axis represents values of *N*. The y-axis shows the F-measure with *N*. **Fig. S11.** Balanced accuracy of CMIC trained with short CGIs for long CGIs. The x-axis indicates the range of CGI sequence lengths. The y-axis indicates the F-measure. **Fig. S12.** MCC of CMIC trained with short CGIs for long CGIs. The x-axis indicates the range of CGI sequence lengths. The y-axis indicates the F-measure. **Fig. S13.** AUC of CMIC trained with short CGIs for long CGIs. The x-axis indicates the range of CGI sequence lengths. The y-axis indicates the F-measure. **Fig. S14.** Balanced accuracy of CMIC with alternative recurrent units, RNN, BiRNN, GRU, BiGRU, LSTM, and BiLSTM. **Fig. S15.** MCC of CMIC with alternative recurrent units, RNN, BiRNN, GRU, BiGRU, LSTM, and BiLSTM. **Fig. S16.** AUC of CMIC with alternative recurrent units, RNN, BiRNN, GRU, BiGRU, LSTM, and BiLSTM. **Fig. S17.** Balanced accuracy of KEGRU with various lengths of *k*-mers and strides. The vector size is set to 20. The x-axis represents a pair of *k* and stride. **Fig. S18.** MCC of KEGRU with various lengths of *k*-mers and strides. The vector size is set to 20. The x-axis represents a pair of *k* and stride. **Fig. S19.** AUC of KEGRU with various lengths of *k*-mers and strides. The vector size is set to 20. The x-axis represents a pair of *k* and stride. **Fig. S20.** Balanced accuracy of KEGRU with various lengths of *k*-mers and strides. The vector size is set to 50. The x-axis represents a pair of *k* and stride. **Fig. S21.** MCC of KEGRU with various lengths of *k*-mers and strides. The vector size is set to 50. The x-axis represents a pair of *k* and stride. **Fig. S22.** AUC of KEGRU with various lengths of *k*-mers and strides. The vector size is set to 50. The x-axis represents a pair of *k* and stride. **Fig. S23.** Plot generated by t-SNE with perplexity 10. **Fig. S24.** Plot generated by t-SNE with perplexity 50.

## Data Availability

CMIC is available through the GitHub at https://github.com/maruyama-lab-design/CMIC under the GNU General Public License v3. The WGBS datasets of FGO (reprocessed for this study) and blastocyst (generated during this study) are available in the NCBI's Gene Expression Omnibus and the DDBJ Sequence Read Archive under accession numbers GSE112320, GSE174311, DRA000570, DRA005849 and DRA011758.

## Abbreviations

CGI: CpG Island
GRU: Gated recurrent unit
RNN: Recurrent neural network
LSTM: Long short-term memory
FGO: Fully grown oocyte
WGBS: Whole-genome bisulfite sequencing
