# Supplementary material for: CMIC: predicting DNA methylation inheritance of CpG islands with embedding vectors of variable-length k-mers
Source: BMC Bioinformatics. 2022 Sep 12;23:371. doi: 10.1186/s12859-022-04916-3 (PMC9469632; doi:10.1186/s12859-022-04916-3)
Supplement: Supplementary file 1 — Additional file 1. Fig. S1. Distribution of methylation ratios in the maternal genome of mouse blastocyst of the CGIs that are methylated in FGOs. The lower bound of class M (methylated) for FGOs is set to βM = 0.8 as shown in Table. 2. Fig. S2. Balanced accuracy of CMIC with different pairs of kmin and kmax. The search space of the pairs of kmin and kmax are set to be kmin = 2, . . . , 11 and kmax = 3, . . . , 12 with kmax − kmin ≥ 1. The bars are grouped by kmax. Fig. S3. MCC of CMIC with different pairs of kmin and kmax. The search space of the pairs of kmin and kmax are set to be kmin = 2, ..., 11 and kmax = 3, ..., 12 with kmax − kmin ≥ 1. The bars are grouped by kmax. Fig. S4. AUC of CMIC with different pairs of kmin and kmax. The search space of the pairs of kmin and kmax are set to be kmin = 2, ..., 11 and kmax = 3, ..., 12 with kmax − kmin ≥ 1. The bars are grouped by kmax. Fig. S5. Balanced accuracy with different schemes for embedding vectors shown in Table 3. The x-axis represents the eight methods to be compared: splitDNA2vec-C, splitDNA2vec-V, splitDNA2vec-sh-C, splitDNA2vec-sh-V, dna2vec-C, dna2vec-V, dna2vec-N1000-C, and dna2vec-N1000-V. The y-axis shows the F-measure on test datasets. Fig. S6. MCC with different schemes for embedding vectors shown in Table 3. The x-axis represents the eight methods to be compared: splitDNA2vec-C, splitDNA2vec-V, splitDNA2vec-sh-C, splitDNA2vec-sh-V, dna2vec-C, dna2vec-V, dna2vec-N1000-C, and dna2vec-N1000-V. The y-axis shows the F-measure on test datasets. Fig. S7. AUC with different schemes for embedding vectors shown in Table 3. The x-axis represents the eight methods to be compared: splitDNA2vec-C, splitDNA2vec-V, splitDNA2vec-sh-C, splitDNA2vec-sh-V, dna2vec-C, dna2vec-V, dna2vec-N1000-C, and dna2vec-N1000-V. The y-axis shows the F-measure on test datasets. Fig. S8. Balanced accuracy of CMIC with different numbers of variable-length k-mer sequences generated from an input CGI sequence, N. The x-axis represents v [file 12859_2022_4916_MOESM1_ESM.pdf]

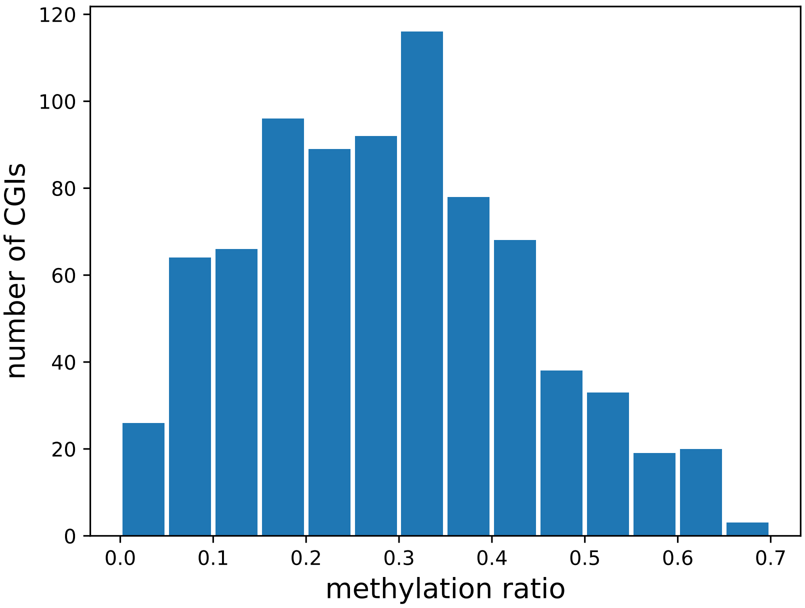

Additional Fig. S1. Distribution of methylation ratios in the maternal genome of mouse blastocyst of the CGIs that are methylated in FGOs. The lower bound of class `M` (methylated) for FGOs is set to $\beta_M = 0.8$ as shown in Table. 2.

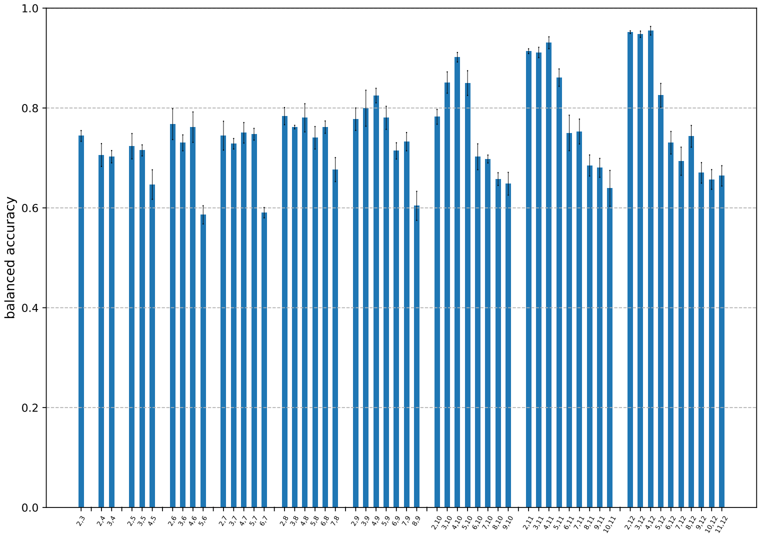

Additional Fig. S2. Balanced accuracy of CMIC with different pairs of $k_{\min}$ and $k_{\max}$. The search space of the pairs of $k_{\min}$ and $k_{\max}$ are set to be $k_{\min} = 2, \ldots, 11$ and $k_{\max} = 3, \ldots, 12$ with $k_{\max} - k_{\min} \geq 1$. The bars are grouped by $k_{\max}$.

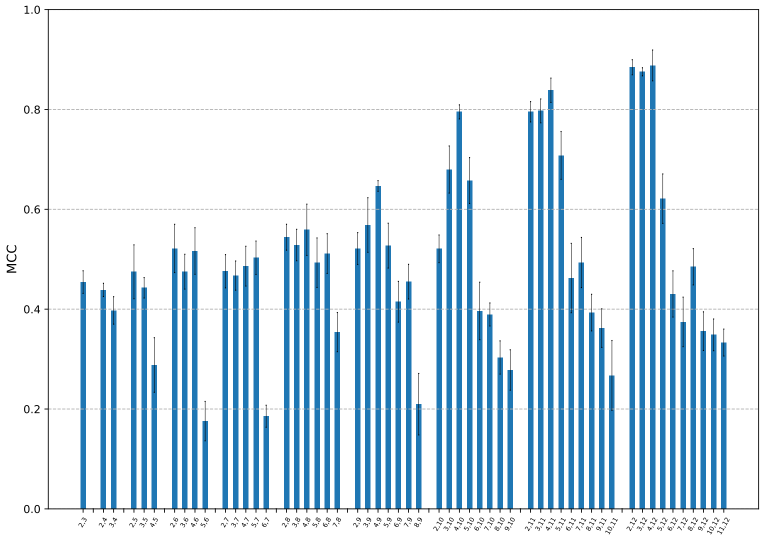

Additional Fig. S3. MCC of CMIC with different pairs of k_{min} and k_{max}. The search space of the pairs of k_{min} and k_{max} are set to be $k_{\text{min}} = 2, \ldots, 11$ and $k_{\text{max}} = 3, \ldots, 12$ with $k_{\text{max}} - k_{\text{min}} \geq 1$. The bars are grouped by k_{max}.

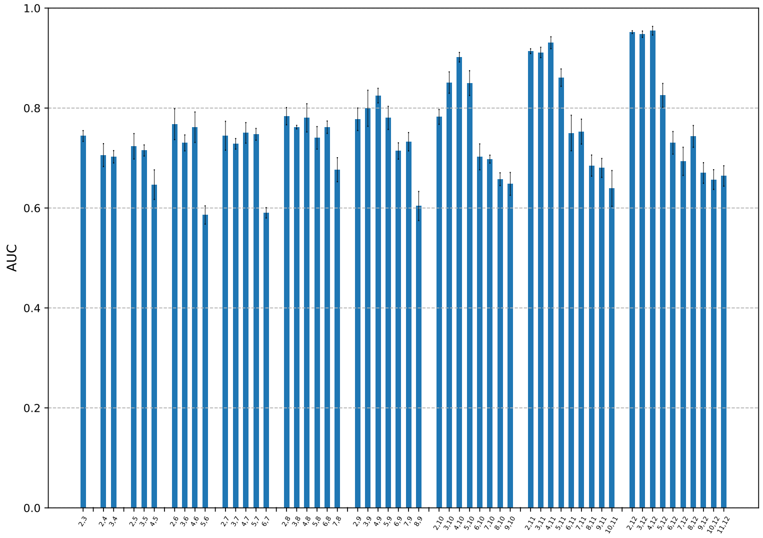

Additional Fig. S4. AUC of CMIC with different pairs of $k_{\min}$ and $k_{\max}$. The search space of the pairs of $k_{\min}$ and $k_{\max}$ are set to be $k_{\min} = 2, \ldots, 11$ and $k_{\max} = 3, \ldots, 12$ with $k_{\max} - k_{\min} \geq 1$. The bars are grouped by $k_{\max}$.

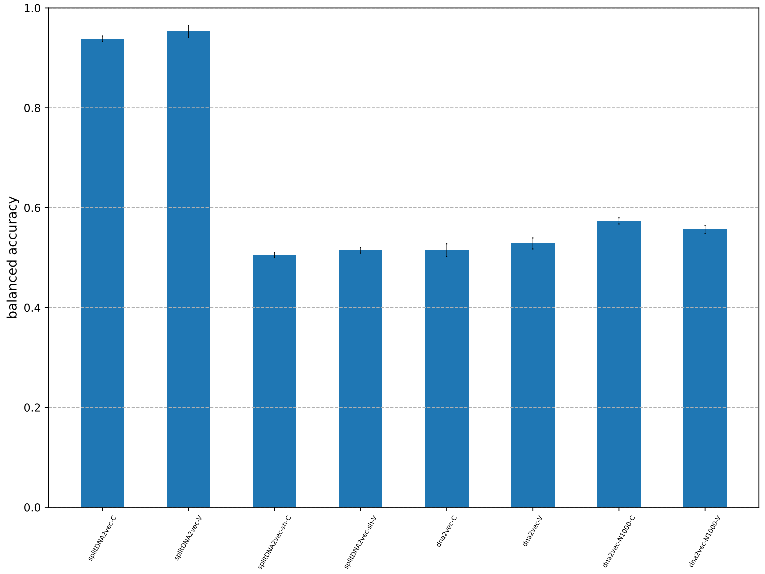

Additional Fig. S5. Balanced accuracy with different schemes for embedding vectors shown in Table 3. The x-axis represents the eight methods to be compared: splitDNA2vec-C, splitDNA2vec-V, splitDNA2vec-sh-C, splitDNA2vec-sh-V, dna2vec-C, dna2vec-V, dna2vec-N1000-C, and dna2vec-N1000-V. The y-axis shows the F-measure on test datasets.

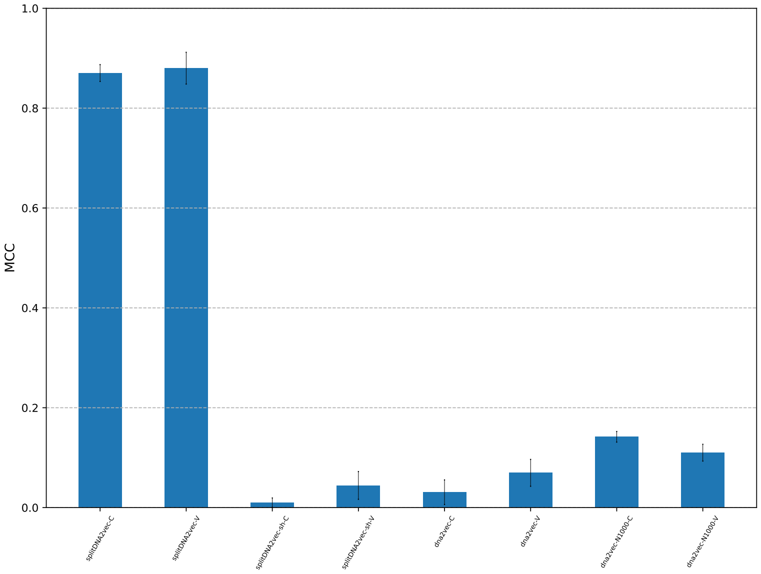

Additional Fig. S6. MCC with different schemes for embedding vectors shown in Table 3. The x-axis represents the eight methods to be compared: splitDNA2vec-C, splitDNA2vec-V, splitDNA2vec-sh-C, splitDNA2vec-sh-V, dna2vec-C, dna2vec-V, dna2vec-N1000-C, and dna2vec-N1000-V. The y-axis shows the F-measure on test datasets.

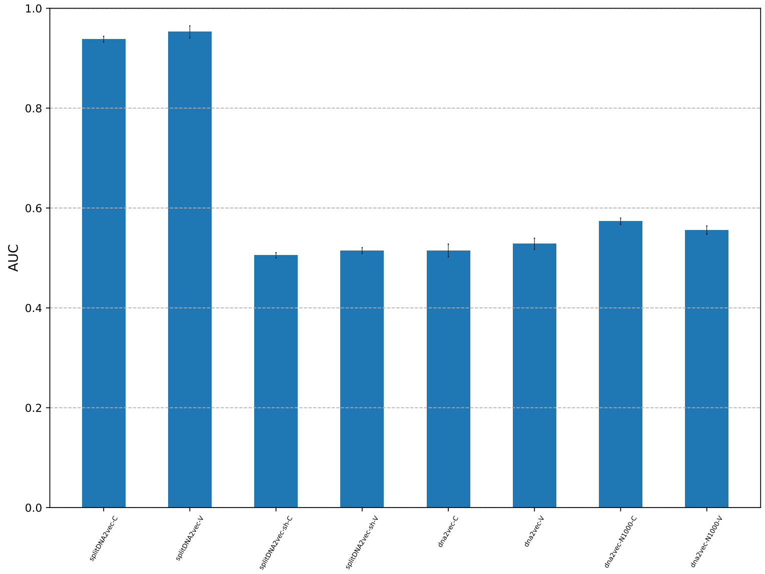

Additional Fig. S7. AUC with different schemes for embedding vectors shown in Table 3. The x-axis represents the eight methods to be compared: splitDNA2vec-C, splitDNA2vec-V, splitDNA2vec-sh-C, splitDNA2vec-sh-V, dna2vec-C, dna2vec-V, dna2vec-N1000-C, and dna2vec-N1000-V. The y-axis shows the F-measure on test datasets.

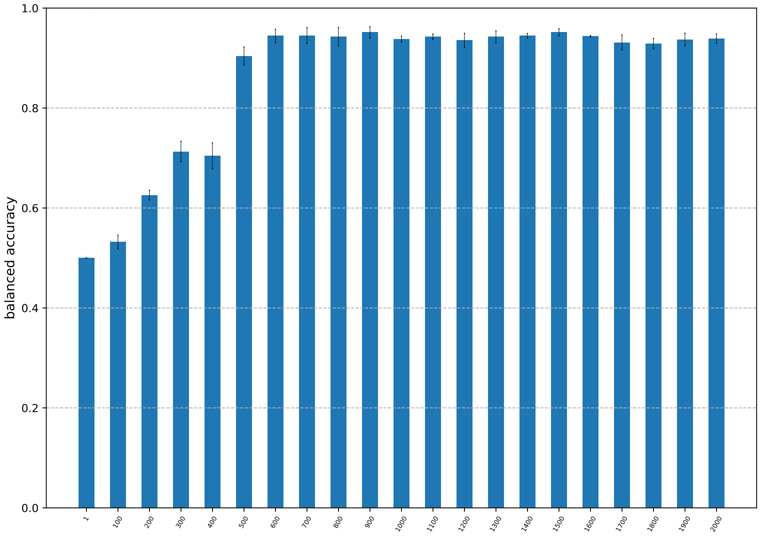

Additional Fig. S8. Balanced accuracy of CMIC with different numbers of variable-length k-mer sequences generated from an input CGI sequence, N. The x-axis represents values of N. The y-axis shows the F-measure with N.

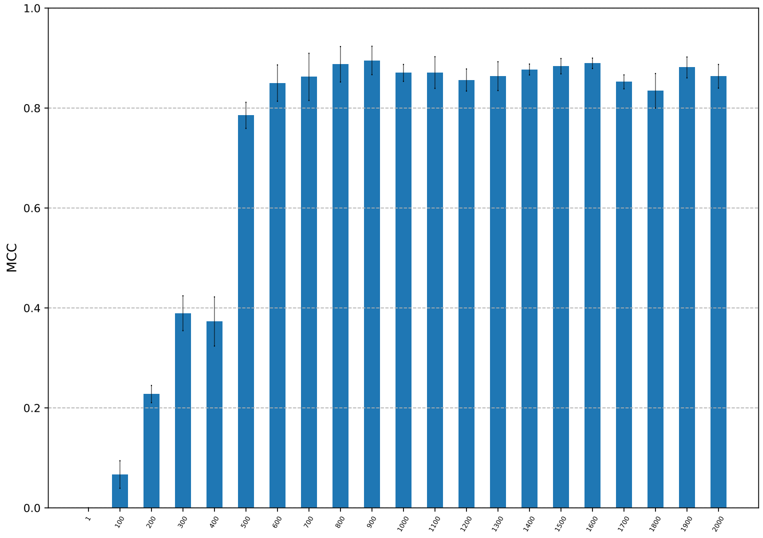

Additional Fig. S9. MCC of CMIC with different numbers of variable-length k-mer sequences generated from an input CGI sequence, N. The x-axis represents values of N. The y-axis shows the F-measure with N.

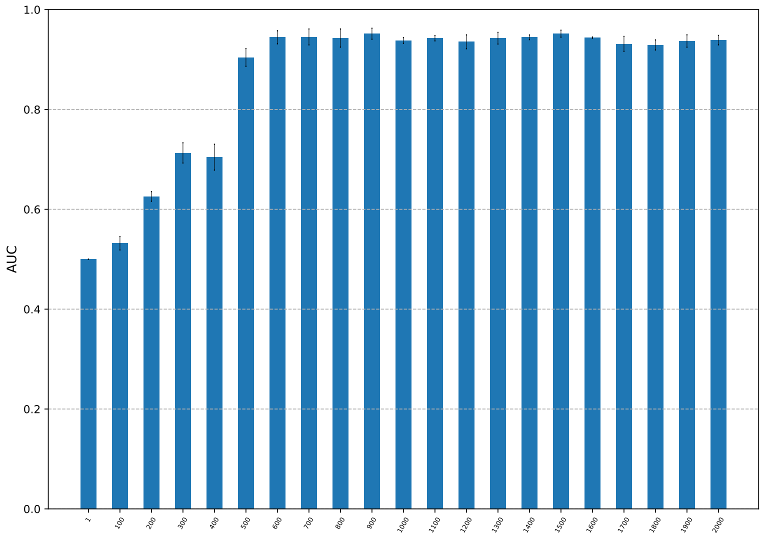

Additional Fig. S10. AUC of CMIC with different numbers of variable-length k-mer sequences generated from an input CGI sequence, N. The x-axis represents values of N. The y-axis shows the F-measure with N.

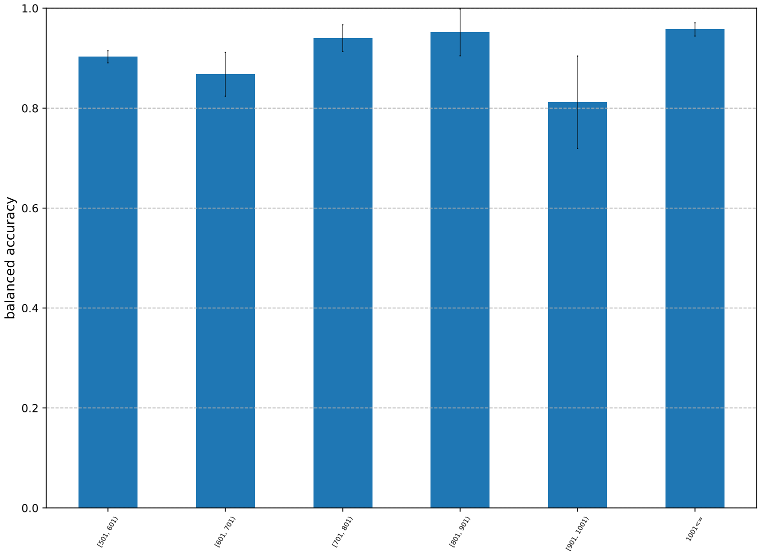

Additional Fig. S11. Balanced accuracy of CMIC trained with short CGIs for long CGIs. The x-axis indicates the range of CGI sequence lengths. The y-axis indicates the F-measure.

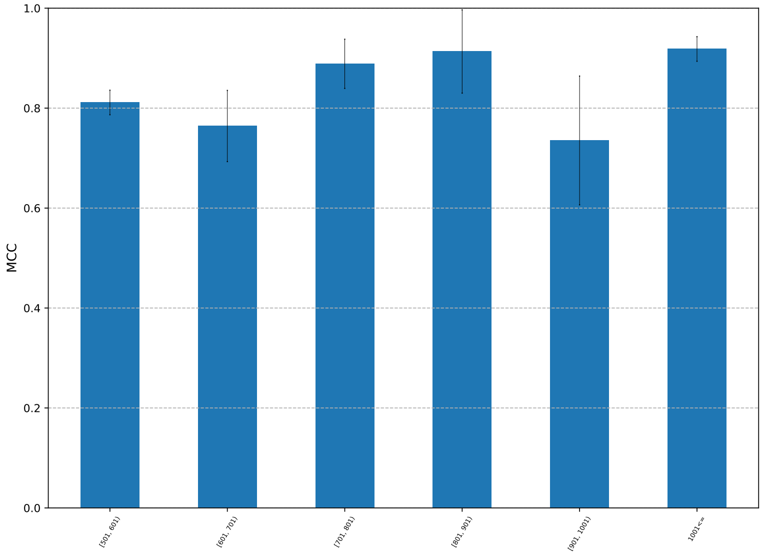

Additional Fig. S12. MCC of CMIC trained with short CGIs for long CGIs. The x-axis indicates the range of CGI sequence lengths. The y-axis indicates the F-measure.

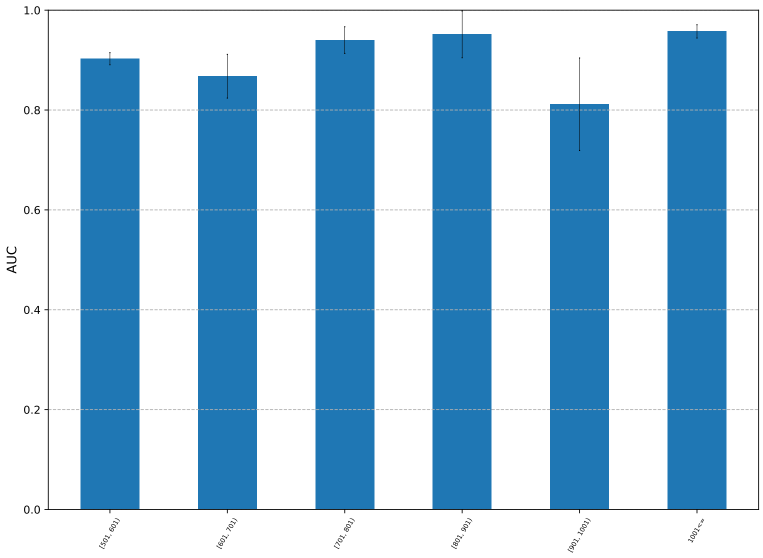

Additional Fig. S13. AUC of CMIC trained with short CGIs for long CGIs. The x-axis indicates the range of CGI sequence lengths. The y-axis indicates the F-measure.

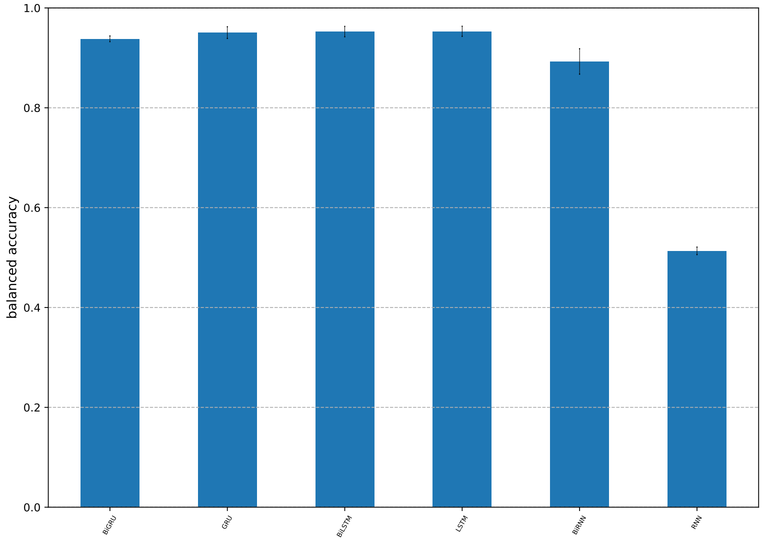

Additional Fig. S14. Balanced accuracy of CMIC with alternative recurrent units, RNN, BiRNN, GRU, BiGRU, LSTM, and BiLSTM.

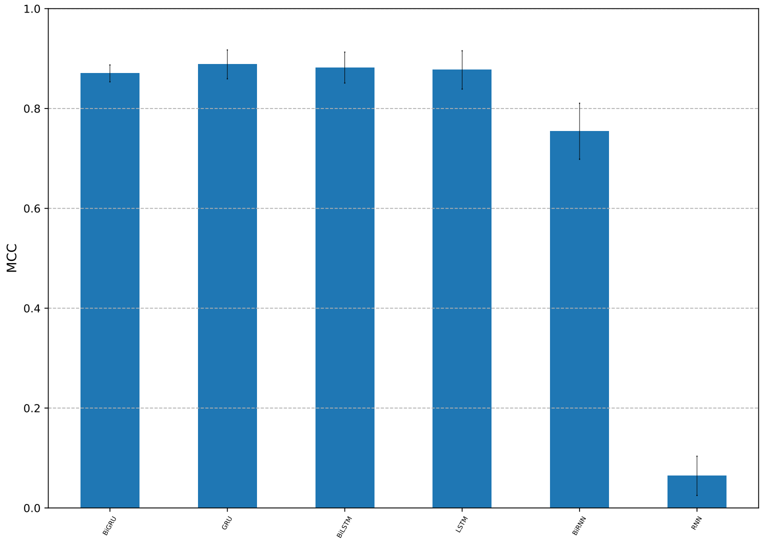

Additional Fig. S15. MCC of CMIC with alternative recurrent units, RNN, BiRNN, GRU, BiGRU, LSTM, and BiLSTM.

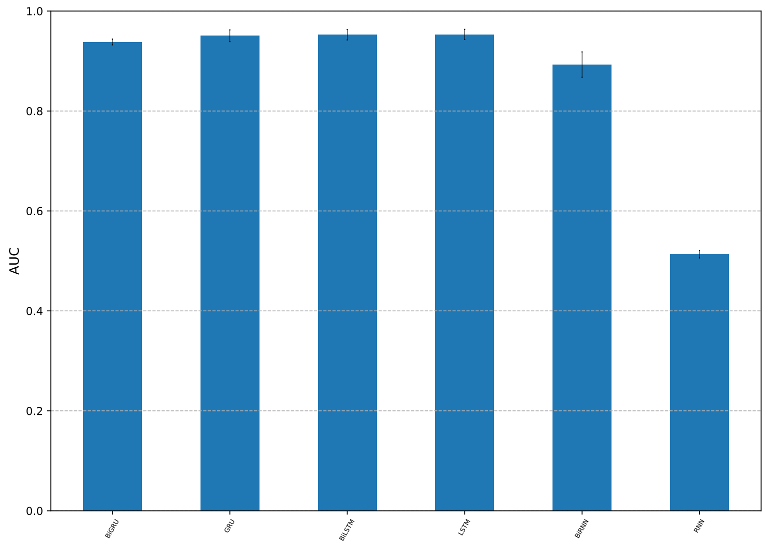

Additional Fig. S16. AUC of CMIC with alternative recurrent units, RNN, BiRNN, GRU, BiGRU, LSTM, and BiLSTM.

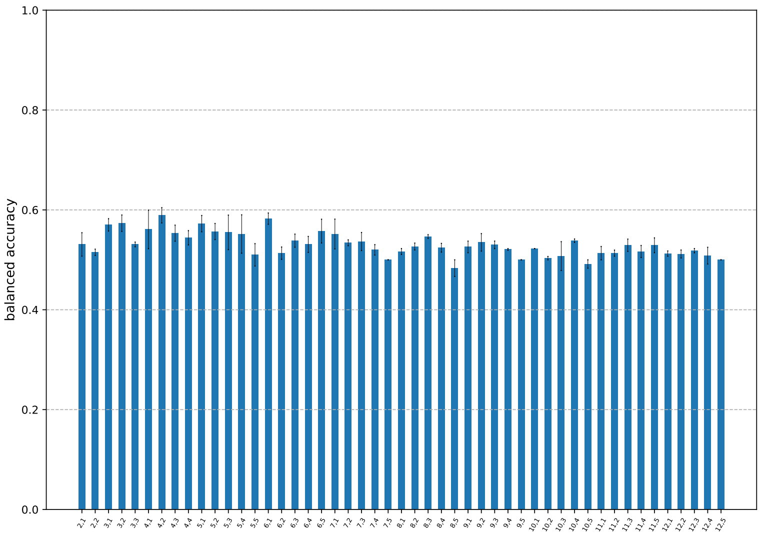

Additional Fig. S17. Balanced accuracy of KEGRU with various lengths of k-mers and strides. The vector size is set to 20. The x-axis represents a pair of k and stride.

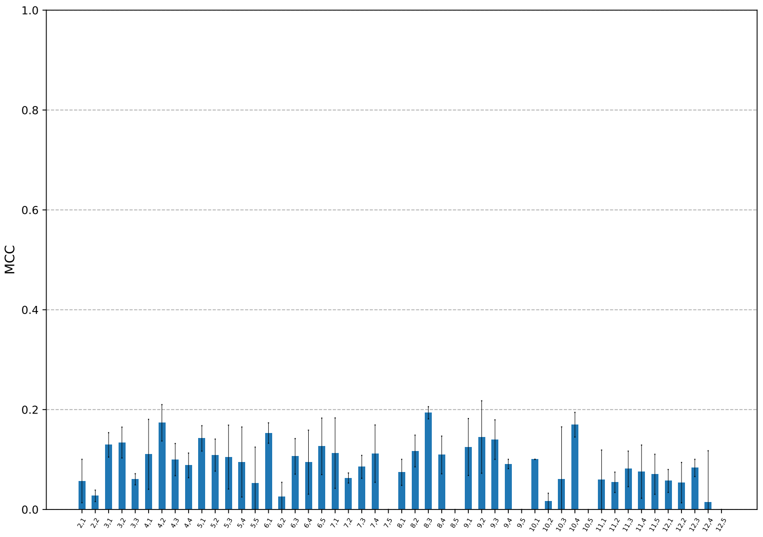

Additional Fig. S18. MCC of KEGRU with various lengths of k-mers and strides. The vector size is set to 20. The x-axis represents a pair of k and stride.

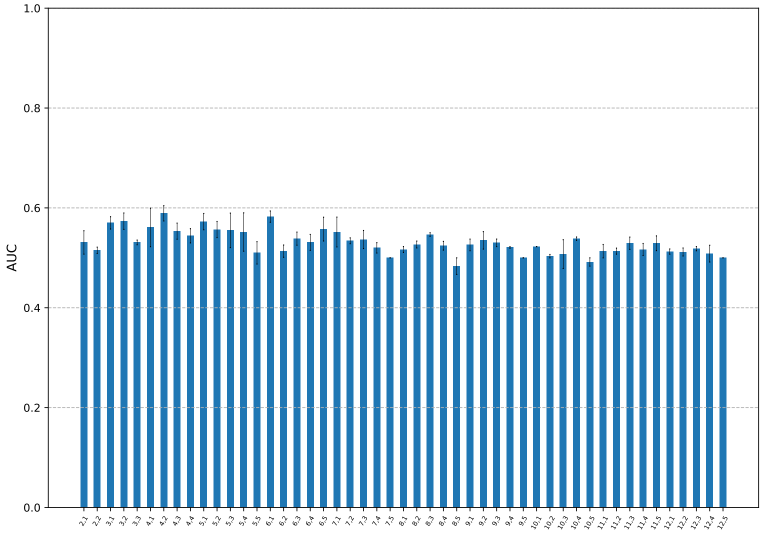

Additional Fig. S19. AUC of KEGRU with various lengths of k-mers and strides. The vector size is set to 20. The x-axis represents a pair of k and stride.

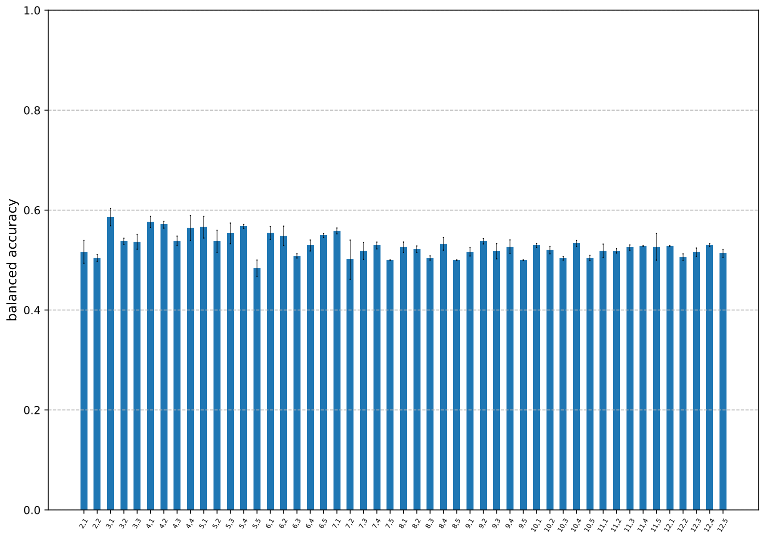

Additional Fig. S20. Balanced accuracy of KEGRU with various lengths of k-mers and strides. The vector size is set to 50. The x-axis represents a pair of k and stride.

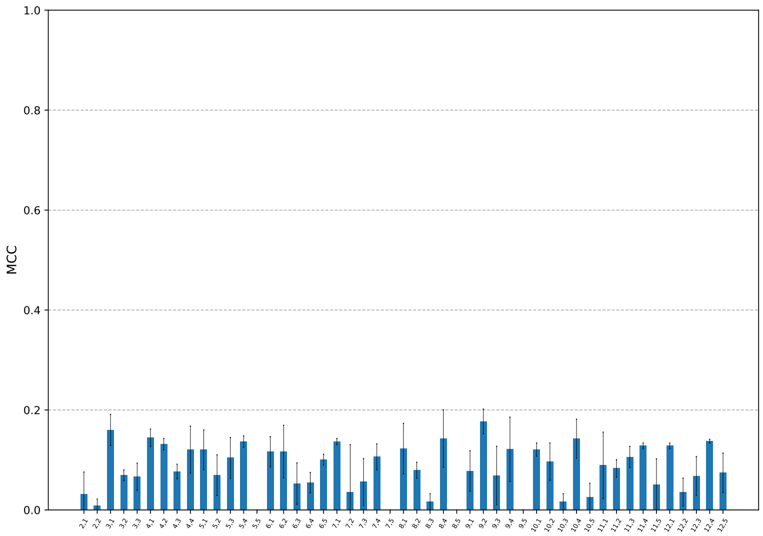

Additional Fig. S21. MCC of KEGRU with various lengths of k-mers and strides. The vector size is set to 50. The x-axis represents a pair of k and stride.

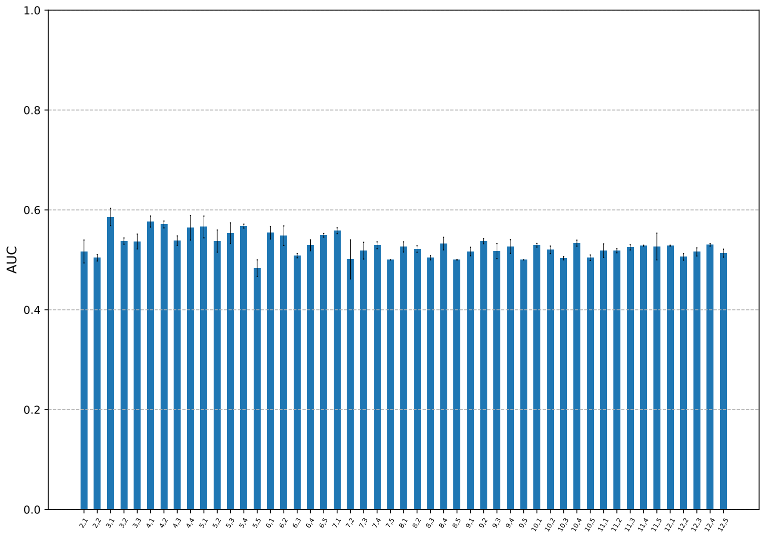

Additional Fig. S22. AUC of KEGRU with various lengths of k-mers and strides. The vector size is set to 50. The x-axis represents a pair of k and stride.

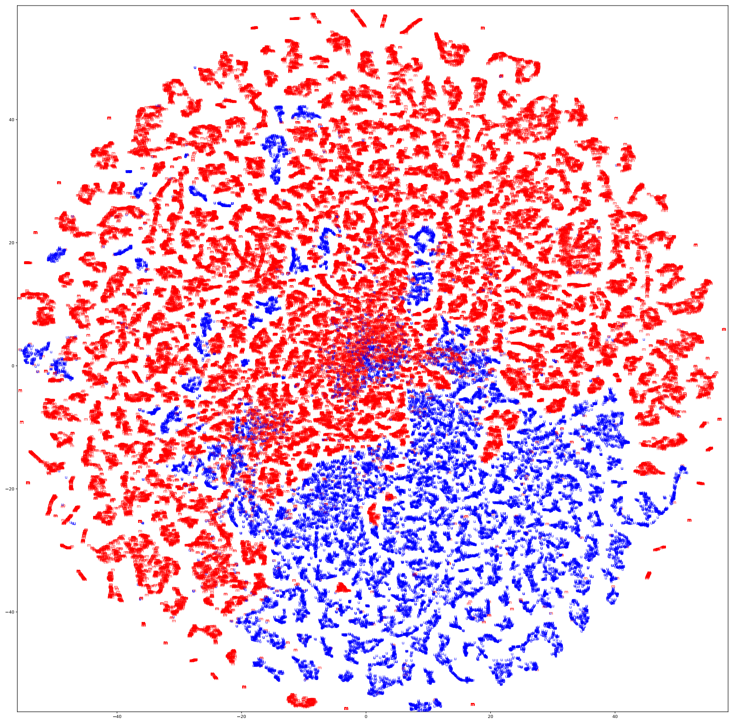

Additional Fig. S23. Plot generated by t-SNE with perplexity 10.

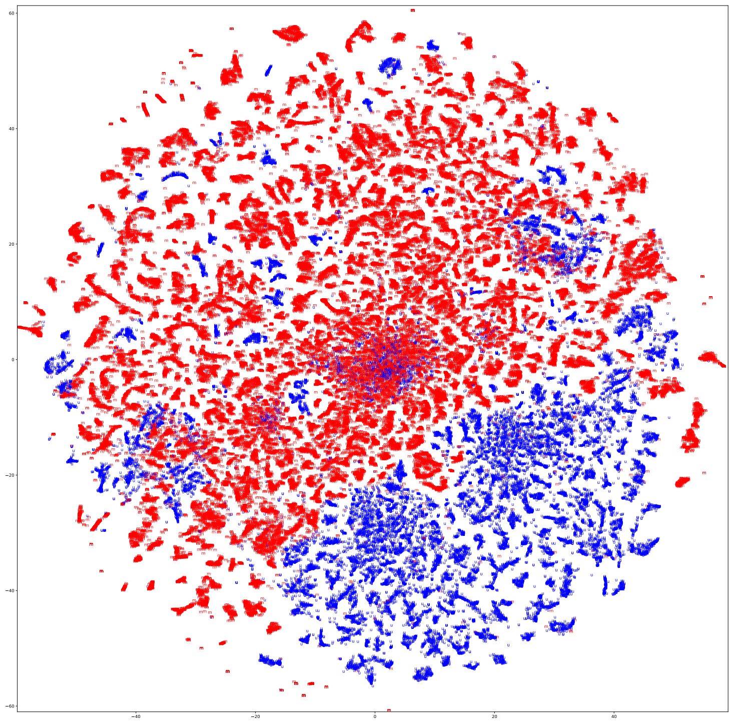

Additional Fig. S24. Plot generated by t-SNE with perplexity 50.
